# Restoring GABA_*B*_ receptor expression in the ventral tegmental area of methamphetamine addicted mice inhibits locomotor sensitization and drug seeking behavior

**DOI:** 10.3389/fnmol.2024.1347228

**Published:** 2024-02-07

**Authors:** Mohammad Hleihil, Dietmar Benke

**Affiliations:** ^1^Institute of Pharmacology and Toxicology, University of Zurich, Zürich, Switzerland; ^2^Neuroscience Center Zurich, University and ETH Zurich, Zürich, Switzerland

**Keywords:** addiction, GABA receptor, interfering peptide, methamphetamine, protein phosphatase 2A, ventral tegmental area (VTA)

## Abstract

Repeated exposure to psychostimulants such as methamphetamine (METH) induces neuronal adaptations in the mesocorticolimbic dopamine system, including the ventral tegmental area (VTA). These changes lead to persistently enhanced neuronal activity causing increased dopamine release and addictive phenotypes. A factor contributing to increased dopaminergic activity in this system appears to be reduced GABA_*B*_ receptor-mediated neuronal inhibition in the VTA. Dephosphorylation of serine 783 (Ser783) of the GABA_*B*2_ subunit by protein phosphatase 2A (PP2A) appears to trigger the downregulation GABA_*B*_ receptors in psychostimulant-addicted rodents. Therefore, preventing the interaction of GABA_*B*_ receptors with PP2A using an interfering peptide is a promising strategy to restore GABA_*B*_ receptor-mediated neuronal inhibition. We have previously developed an interfering peptide (PP2A-Pep) that inhibits the GABA_*B*_ receptors/PP2A interaction and thereby restores receptor expression under pathological conditions. Here, we tested the hypothesis that restoration of GABA_*B*_ receptor expression in the VTA of METH addicted mice reduce addictive phenotypes. We found that the expression of GABA_*B*_ receptors was significantly reduced in the VTA and nucleus accumbens but not in the hippocampus and somatosensory cortex of METH-addicted mice. Infusion of PP2A-Pep into the VTA of METH-addicted mice restored GABA_*B*_ receptor expression in the VTA and inhibited METH-induced locomotor sensitization as assessed in the open field test. Moreover, administration of PP2A-Pep into the VTA also reduced drug seeking behavior in the conditioned place preference test. These observations underscore the importance of VTA GABA_*B*_ receptors in controlling addictive phenotypes. Furthermore, this study illustrates the value of interfering peptides targeting diseases-related protein-protein interactions as an alternative approach for a potential development of selective therapeutic interventions.

## Introduction

Drug addiction is a complex chronic disease with biological, behavioral, and social components. Psychostimulants such as cocaine, amphetamine, and methamphetamine (METH) are highly addictive drugs, whose abuse can lead to serious health problems and significant healthcare costs (May et al., 2020; Cheron and Kerchove d’Exaerde, 2021). For instance, in 2018, 2.3 million employees receiving employer-sponsored insurance in the USA (representing 1.4% of the total population of employees receiving employer-sponsored insurance in the USA) were diagnosed with substance use disorders with total annual medical cost of $35.3 billion (Li M. et al., 2023). Currently, there is no effective pharmacological therapy available to cure patients from addiction.

Addiction to psychostimulants results from repeated and prolonged actions of the drug on the brain inducing long-lasting plastic neuronal adaptations (Ostroumov and Dani, 2018; Fischer et al., 2021). The effects of psychostimulants are mediated via enhanced dopamine release in the mesocorticolimbic dopamine pathway. The mesocorticolimbic circuit is involved in many cognitive processes including reward, motivation, learning, and fear (Volman et al., 2013; Baik, 2020). A key structure triggering addictive behavior is the ventral tegmental area (VTA) located in the midbrain. Acute effects of psychostimulants, such as feelings of euphoria, pleasure, and reward, are mediated via increased activity of VTA dopamine neurons projecting to the nucleus accumbens, amygdala, and medial prefrontal cortex (Nestler, 2005). Repeated and chronic consumption of psychostimulants induces plastic neuronal adaptations resulting in permanently increased dopamine release in the mesocorticolimbic dopamine pathway.

One important factor controlling neuronal excitability and neurotransmitter release is the GABA_*B*_ receptor, which is expressed in most neurons at axon terminals, dendritic and somatic sites (Chalifoux and Carter, 2011). GABA_*B*_ receptors are heterodimeric assemblies composed of GABA_*B*1_ and GABA_*B*2_ subunits and mediate their effects by activating Gi/o proteins (Jones et al., 1998; Kaupmann et al., 1998; White et al., 1998). Their most prominent actions in the brain are the activation of G protein-coupled inwardly rectifying potassium channels (GIRK or K_*ir*_3) at postsynaptic locations to reduce neuronal excitability (Gähwiler and Brown, 1985; Luscher et al., 1997) and the inhibition of voltage-gated Ca^2+^ channels at presynaptic sites to reduce transmitter release (Mintz and Bean, 1993; Santos et al., 1995; Chen and van den Pol, 1998).

GABA_*B*_ receptors are well expressed in the mesocorticolimbic dopamine system, where they control neuronal activity and the release of GABA, glutamate and dopamine (Lalive and Lüscher, 2016). Accordingly, pharmacological activation of GABA_*B*_ receptors by the selective agonist baclofen reduced several addictive behaviors in preclinical studies such as the development and expression of psychostimulant-induced motor sensitization (Bartoletti et al., 2004, 2005; Cedillo et al., 2019), drug seeking (Li et al., 2001), drug self-administration (Roberts and Brebner, 2000; Ranaldi and Poeggel, 2002; Brebner et al., 2005) and drug-induced cognitive impairment (Arai et al., 2009; Mizoguchi and Yamada, 2011). Early clinical studies reported some positive effects of baclofen in reducing psychostimulant consumption and drug craving (Ling et al., 1998; Shoptaw et al., 2003; Heinzerling et al., 2006). There are clinical evidences for the benefit of baclofen for the treatment of alcohol use disorders (Agabio et al., 2023), but its use is controversial (de Beaurepaire et al., 2018; Leggio and Litten, 2021). Currently, baclofen is approved for the treatment of alcohol addiction in France (Garbutt, 2020; Rolland et al., 2020). One problem of baclofen at high dosage are numerous side effects such as sedation, dizziness, fatigue, insomnia, headache, paresthesia, tinnitus, and restlessness (Rolland et al., 2018). The wide range of adverse side effects might reflect the fact that systemic application of baclofen activates all GABA_*B*_ receptors in the body and not only in the neuronal pathways related to the addicted state. This might generally restrict the broad clinical application of baclofen and need for more specific interventions.

There is considerable evidence that GABA_*B*_ receptor-mediated inhibition is reduced in brain structures of the mesocorticolimbic system after psychostimulant treatment, diminishing inhibitory control and enhancing neuronal excitability (Arora et al., 2011; Padgett et al., 2012; Hearing et al., 2013; Sharpe et al., 2015; Munoz et al., 2016). This has been attributed to downregulation of either GABA_*B*_ receptors and GIRK channels or both (Arora et al., 2011; Padgett et al., 2012; Hearing et al., 2013; Munoz et al., 2016; Lu et al., 2023). Aberrant downregulation of GABA_*B*_ receptors is a response to sustained activity of glutamate receptors, which dysregulates GABA_*B*_ receptor trafficking from the constitutive recycling pathway to lysosomal degradation (Guetg et al., 2010; Maier et al., 2010; Terunuma et al., 2010). Key steps in this aberrant regulation are the phosphorylation of Ser867 in the GABA_*B*1_ subunit mediated by Ca^2+^-calmodulin dependent protein kinase IIβ (CaMKIIβ) (Guetg et al., 2010; Zemoura et al., 2019) and dephosphorylation of Ser783 in GABA_*B*2_ by protein phosphatase 2A (PP2A) (Terunuma et al., 2010; Hleihil et al., 2022). Downregulation of cell surface and total GABA_*B*_ receptors in the nucleus accumbens after repeated cocaine administration involved the activation of CaMKII (Lu et al., 2023), whereas selective loss of cell surface receptors was observed in the mPFC and VTA after cocaine and methamphetamine exposure, respectively, due to PP2A-mediated dephosphorylation of Ser783 in GABA_*B*2_ (Padgett et al., 2012; Hearing et al., 2013).

Recently, we developed an interfering peptide (PP2A-Pep) that inhibits the interaction of GABA_*B*_ receptors with PP2A (Hleihil et al., 2022). This peptide restored downregulated GABA_*B*_ receptor expression after an ischemic insult, normalized neuronal overexcitation and inhibited progressive neuronal death. Since GABA_*B*_ receptor expression and function appears to be reduced in the VTA after METH application, we tested in the present study the hypothesis whether restoring GABA_*B*_ receptor expression in the VTA of METH-addicted mice, by blocking the interaction of GABA_*B*_ receptors with PP2A, would reduce phenotypes associated with addiction. For this we infused PP2A-Pep into the VTA of METH-addicted mice and tested for GABA_*B*_ receptor expression using immunohistochemistry, locomotor sensitization in the open field test (OFT) and drug seeking behavior in the conditioned place preference test (CPP).

## Materials and methods

### Animals

Experiments were performed using 8–10 weeks old adult male and female C57BL/6 mice. Mice were either purchased from Envigo (Netherlands) or bred at the Laboratory Animal Service Center of the University of Zurich (Switzerland). The mice were group-housed up to five in filter-top cages with a standard 12/12-h light/dark cycle and food and water available *ad libitum*. All experiments were approved by the veterinary office of the Canton of Zurich (license ZH164/2020).

### Interfering peptide (PP2A-Pep)

The interfering peptide (PP2A-Pep, YTIWMPENPRPGTP CDIFTNSRGKRASNGGGGRRRRRRRRRFQFTQNQKKEDSKTS TSV) used in this study inhibits the interaction of PP2A with GABA_*B*_ receptors (Hleihil et al., 2022). It contains a sequence derived from the intracellularly located C-terminal domain of GABA_*B*2_ (red characters) and was rendered cell permeable by tagging it at the N-terminus with a peptide sequence derived from the Rabies virus glycoprotein followed by 9 arginine residues (gray characters). An inactive control peptide (Ctrl-Pep) contained the same amino acids but in a random sequence (except for the cell penetrating peptide sequence, YTIWMPENPRPGTPCDIFTNSRGKRASNGGGGRRRRRRRRRQ KFSVNTFQEKDTKSQTS). The peptides were additionally tagged with biotin to permit their detection by immunohistochemical staining using AlexaFluor 488-conjugated streptavidin. The peptides were custom-synthesized by Pepmic Co., Ltd., Suzhou, China.

### Cannulation and peptide administration

Cannula implantations were conducted using a stereotaxic frame (David Kopf Instruments and NeuroStar) under general anesthesia using continuous isoflurane inhalation via a nose mask (induction: 5% at 1 l/min, surgery: 1.5% in a mixture of O_2_ and air at 0.4–0.6 l/min). The body temperature of the mice was kept at 36.5 ± 0.5°C using a feed-back controlled heating mat with a rectal temperature probe. Eyes were protected from drying using vitamin A ointment. For analgesia, mice received a subcutaneous injection of buprenorphine (0.1 mg/kg) 20–30 min before surgery and an additional subcutaneous injection of lidocaine (2 mg/kg)/bupivacaine (1 mg/kg) at the site of incision about 5 min before surgery. Before exposing the skull, the incision site was cleaned with 70% ethanol. A unilateral cannula implantation was performed using a 26G guide cannula (#C315GS-5/SPC, Bilaney Consultants, Germany). A hole was drilled through the skull above the VTA (anterior-posterior −3.3; medial-lateral −0.9, relative to bregma). The guide cannula was placed through the hole above the VTA with a 10° angle (dorsal-ventral −4.25). The cannula was held in place using dental cement. All mice received post-surgical analgesia for additional 2 days (10 mg/kg carprofen, s. c. twice per day).

Peptides (0.8 μl PP2A-Pep, 100 μg/ml or 0.8 μl Ctrl-Pep, 100 μg/ml) were administered into the VTA with a rate of 100 nl/min using an automatic pump (PHD ULTRA Nanomite, Harvard Apparatus) connected by PE tubing which was attached to an injection cannula (#C315DCS-4/SPC, Bilaney Consultants, Germany) with 0.25 mm projection from the tip of the guiding cannula. Following injections, the cannula was left in place for additional 5 min to allow peptide infusion.

### Behavioral tests

Seven days prior to any experiment, the mice were habituated to the experimenter by gentle handling in the housing facility. At least 1 h prior to the experiment, mice were transferred to the experimental room (50 lux dim light). All tests were performed in the light cycle. The respective experimental apparatus was cleaned with 70% ethanol before testing a mouse to avoid any sensory stimulation related to the previous mouse tested. In this study we used non-contingent drug administration as it ensures the expression of robust addictive phenotypes.

#### Open field test (OFT)

The OFT was used to test for METH-induced locomotor sensitization (Good and Radcliffe, 2011; Jing et al., 2014). The locomotor activity was monitored in the open-field test chamber (32 cm × 32 cm × 23 cm) with an infrared detection system under dim light conditions (50 lux). Mice were transferred from the housing facility to the experimental room 1 h prior to the experiment. All mice received i.p. injection (saline or METH) and immediately placed into the open field chamber (32 cm × 32 cm × 23 cm), where their activity was recorded for 30 min. Then the mice were returned to their home cages.

To induce methamphetamine (METH) addiction, mice were injected alternatively with either saline or methamphetamine (METH, 2 mg/kg, i. p., dissolved in saline) on every other day for 10 days (METH injections on day 1, 3, 5, 7 and 9; saline injections on day 2, 4, 6, 8, 10). Even a single METH injection has been shown to induce robust and long-lasting locomotor sensitization (Jing et al., 2014). We verified the development of locomotor sensitization in a subgroup of mice by daily open field tests. The control group received solely saline injections for 10 days. One day after the last saline injection (day 11), mice were microinfused with the peptides dissolved in saline (0.8 μl PP2A-Pep, 100 μg/ml or 0.8 μl Ctrl-Pep, 100 μg/ml) via the implanted cannula and then placed back to their home cage. The next day (day 12), the mice were injected with a challenging dose of METH (1 mg/kg, i. p., dissolved in saline) and immediately placed into the open-field test chamber to allow free exploration for 30 min. The mice were monitored by a digital camera placed above the center of the arena. From each session, 10 min of the recordings were extracted for analysis with the EthoVision XT 15 software (Noldus, Wageningen, Netherlands).

#### Conditioned place preference (CPP)

CCP was used to test drug seeking behavior (Cunningham et al., 2006). CPP was conducted in a two-compartment plexiglass apparatus (size of each compartment: 20 cm × 20 cm × 15 cm), in which the two conditioning compartments were connected through a short tunnel (10 cm) (Figure 5). The two conditioning chambers were visually and tactile distinct. Chamber 1 consisted of white walls with black strips and a plain white floor with gray cross lines. Chamber 2 consisted of black walls and a rough textured plexiglass floor. The behavior of the mice was recorded by a digital camera placed above the CCP device.

The CPP procedure consisted of three phases (Figure 5): habituation and baseline preference (day 1), conditioning phase (days 2–6) and preference tests (days 7 and day 8). In the habituation/preference evaluation (day 1), mice were placed in the tunnel connecting the two chambers and allowed to freely explore all compartments for 30 min. This allowed to assign a preferred compartment for each mouse to conduct a biased CPP experiment: the mice received saline injections in their initially preferred chamber (saline-paired chamber) and METH injections in the non-preferred compartment (METH-paired chamber).

During the conditioning phase (day 2–6), access was restricted to the saline-paired or METH-paired compartment. In the METH group, mice were injected with METH (2 mg/kg, i.p., dissolved in saline) and immediately placed into the METH-paired chamber for 30 min and then returned to the home cage. Six hours later, the mice were injected with saline and placed into the saline-paired chamber for 30 min. For the control saline group, the same procedure was conducted but mice were injected with two saline doses.

On the test day (day 7), the preference test was performed 24 h after the last conditioning trial by placing the mouse in the tunnel connecting the two compartments and allowing to freely explore all chambers for 10 min. One hour after the test, mice were microinfused with the peptides dissolved in saline via the implanted cannula (0.8 μl PP2A-Pep, 100 μg/ml or 0.8 μl Ctrl-Pep, 100 μg/ml) and then placed back into their home cage. The timeline for peptide treatment was selected according to Li J. Y. et al. (2023), which predominantly affects memory reconsolidation.

On day 8, the preference test was conducted again for 10 min to test for the peptide effect. The recordings were analyzed with the EthoVision XT 15 software (Noldus, Wageningen, Netherlands) to extract the CPP data. The CPP preference score was calculated as a difference between the relative amount of time spent in the saline-paired chamber to the time spent in the METH-paired chamber.

### Antibodies

Primary antibodies: rabbit anti-GABA_*B*2*N*_ [1:250, custom-made by GeneScript, (Benke et al., 2002)], rabbit anti-GFAP (1:500, Agilent Technologies # Z0334), guinea pig anti-NeuN (1:500, Synaptic systems # 266004), rabbit anti-Iba1 (1:500, WAKO Chemicals # 019-19741).

Secondary antibodies: donkey anti-rabbit AlexaFluor Plus 555 (1:2000, Thermo Fisher Scientific # A32794), goat anti-guinea pig AlexaFluor 647 (1:1000, Jackson ImmunoResearch # 706-605-148), and AlexaFluor 488-conjugated streptavidin (1:500, Jackson ImmunoResearch # 016-540-084).

### Tissue preparation and immunohistochemistry

A loss of GABA_*B*_ receptor immunofluorescence staining under ischemic conditions always correlated with a similar reduction of receptor protein signals in western blots and with diminished GABA_*B*_ receptor mediated inhibition (see e.g., Zemoura et al., 2019; Hleihil et al., 2021, 2022; Bhat et al., 2022; Balakrishnan et al., 2023). Therefore, we assume that a reduction in GABA_*B*_ receptor immunofluorescence staining in this study indicates diminished GABA_*B*_ receptor mediated inhibition.

Immediately after the last behavioral test, mice were transcranially perfused with ice-cold oxygenated ACSF (125 mM NaCl, 2.5 mM KCl, 1.25 mM NaH_2_PO_4_, 25 mM NaHCO_3_, 1 mM MgCl_2_, 2 mM CaCl_2_, and 25 mM glucose, pH 7.4, pH 7.4), for 2 min. Brains were then immediately dissected and cut in the medial midline. One of the hemispheres were then fixed in 4% PFA overnight, followed by incubation in 30% sucrose/PBS for 24 h at 4°C for immunohistochemical staining.

For immunohistochemistry, free floating 40 μm thick coronal sections were prepared using a sliding microtome (HM400; Microm) and stored in antifreeze solution (50 mM Na-phosphate buffer, pH 7.4, 1.6 M Glucose, 20 mM ethylene glycol, 6 mM sodium azide) at −20°C for further analysis. After rinsing once in PBS, sections were incubated overnight at 4°C with primary antibody (or AlexaFluor 488-conjugated streptavidin for peptide detection) prepared in Tris-Triton solution [50 mM Tris pH 7.4, 150 mM NaCl containing 0.2% Triton X-100 and 5% normal donkey serum (NDS)]. After 3 washes for 10 min with Tris-Triton without NDS, sections were incubated with secondary antibody diluted in Tris-Triton containing 2% NDS for 1 h at room temperature. Following 3 washes for 10 min with Tris-Triton without NDS, brains were mounted onto glass slides (SuperFrost Plus, Thermo Fisher Scientific), dried and coversliped with DAKO fluorescence mounting medium supplemented with DAPI (Fluoroshield with DAPI, #F6057, Merk).

Images were acquired using a LSM 800 confocal microscope (Carl Zeiss). For imaging of the brain areas, a stacks of 10 optical sections at 1 μm z-spacing were taken using a plan-apochromat 10x/0.45 objective in sequential mode with a resolution of 1,024 × 1,024 pixels. The laser intensity and the detector gain were adjusted to values that avoid signal saturation and all images of one experiment was imaged with the same settings in one continuous session. For images on the cellular level, 5 optical sections with 0.7 μm z-spacing were taken using a 40x/1.4 plan-apochromat oil differential interference contrast objective with 0.5x digital zoom.

For quantification of GABA_*B*_ receptor expression, all images belonging to an experiment were recorded with identical settings. Analysis was performed after merging the optical planes into a single image and an area of interest was defined. The mean fluorescence values were determined using ImageJ (Schindelin et al., 2012).

The original images recorded were too dim to be shown directly. Therefore, we enhanced the brightness of the images to make the staining more easily visible to the reader.

### Statistical analysis

The data is shown as means ± SD. Immunohistochemical data was analyzed by two-way analysis of variance with type III sum of square calculations for an unbalanced data design (ANOVA, Figures 2, 3) using GraphPad Prism 8.4.3, followed by Tukey’s multiple comparison test as indicated in the respective figure legends. Behavioral experiments were analyzed by two-way AVOVA with type III sum of square calculations for an unbalanced data design (Figures 4, 5D) and two-tailed unpaired *t*-test (Figure 5C). All data sets passed tests for normal distribution and homoscedasticity. Differences were considered statistically significant when *p* < 0.05.

## Results

### Neuron-specific uptake of PP2A-Pep

**FIGURE 1:**
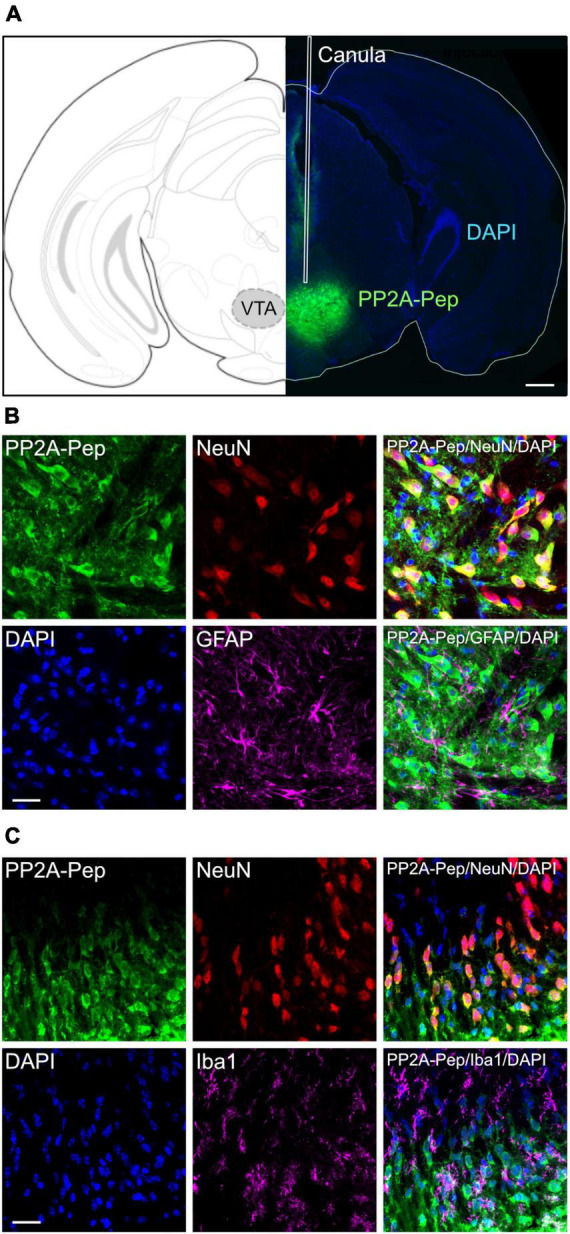
Specific uptake of PP2A-Pep into VTA neurons. **(A)** Infusion site and spread of PP2A-Pep. Right: Brain section stained for the infused PP2A-Pep using AlexaFluor 488-conjugated streptavidin (green) and DAPI (blue). The placement of the canula is indicated (white lines). Left: Location of section in the mouse brain atlas of Paxinos and Franklin (2001). Scale bar: 500 μm. **(B)** PP2A-Pep was selectively taken up by neurons but not by astrocytes. Brain sections containing the VTA were stained for the infused PP2A-Pep (green), Neurons (NeuN, red), astrocytes (GFAP, magenta) and nuclei (DAPI, blue). Scale bar: 20 μm. **(C)** PP2A-Pep was selectively taken up by neurons but not by microglia. Brain sections containing the VTA were stained for the infused PP2A-Pep (green), Neurons (NeuN, red), microglia (Iba1, magenta) and nuclei (DAPI, blue). Scale bar: 20 μm.

For testing the effect of PP2A-Pep on METH-addicted mice, the peptide was unilaterally infused into the VTA via an implanted canula (Figure 1A). Unilateral infusion had been shown to cover both sides of the VTA (Harada et al., 2021). To largely restrict uptake of PP2A-Pep (as well as the control peptide Ctrl-Pep) into neurons, it contained at the N-terminus a cell-penetrating peptide sequence derived from the Rabies virus glycoprotein (Kumar et al., 2007). We previously showed that peptides containing this sequence are selectively taken up by neurons via a receptor-mediated mechanism (Balakrishnan et al., 2023). PP2A-Pep was indeed taken up by neurons after infusion into the VTA as shown by co-immunofluorescence staining for the peptide, neurons (NeuN antibody) and nuclei (DAPI) (Figures 1B, C). The peptide was not taken up by glia cells as shown by co-staining for astrocytes with GFAP antibodies (Figure 1B) and microglia by co-staining with Iba1 antibodies (Figure 1C).

### GABA_*B*_ receptor expression was reduced in the VTA of METH-addicted mice but normalized after PP2A-Pep infusion

**FIGURE 2:**
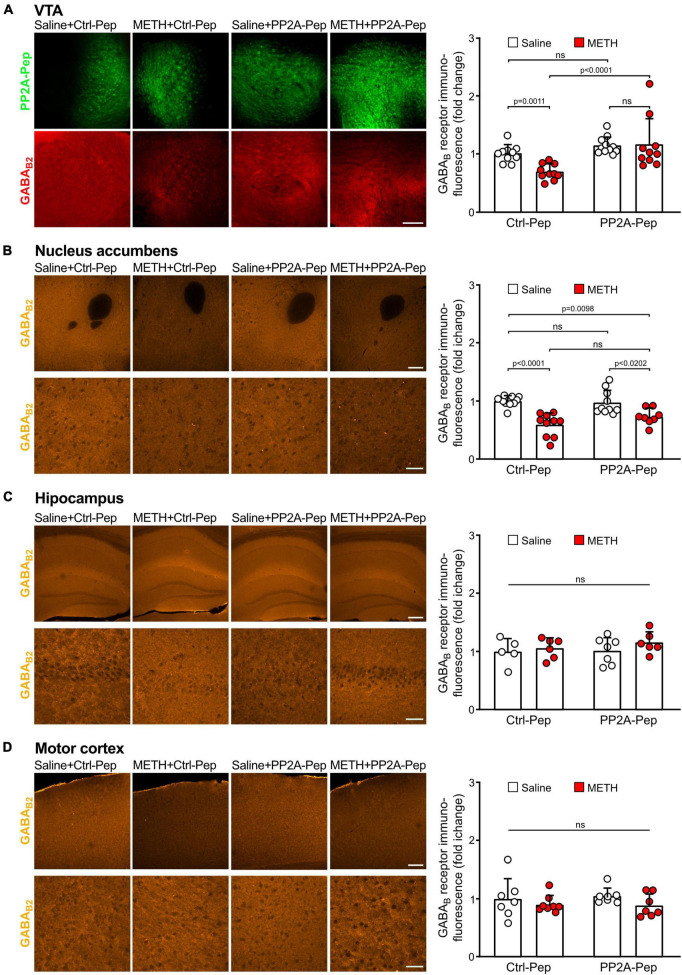
Infusion of PP2A-Pep into the VTA of METH-addicted mice normalized GABA_B_ receptor expression. Mice received multiple METH/saline (METH-addicted mice) or saline/saline (control mice) injections and were then either infused into the VTA with PP2A-Pep or the inactive Ctrl-Pep. About 24 h after peptide application the mice were analyzed for GABA_B_ receptor expression using GABA_B2_ antibodies in the VTA **(A)**, nucleus accumbens **(B)**, hippocampus **(C)**, and motor cortex **(D)** on a regional level. **(A)** GABA_B_ receptor expression was reduced in the VTA of METH-addicted mice and restored after infusion of PP2A-Pep. Left: Representative images display peptide uptake (green) and GABA_B_ receptor expression (red) in the VTA (scale bar: 100 μm). Right: Quantification of fluorescence intensities in the area stained for the peptide (the data was normalized to the mean immunofluorescence value of Ctrl-Pep infused into the VTA of saline treated control mice). The data represents the mean ± SD of 9–10 mice per condition. Statistical analysis: Two-way ANOVA with Tukey’s multiple comparison test (ns, *p* > 0.05). **(B)** GABA_B_ expression was reduced in the nucleus accumbens of METH-addicted mice but unaffected by PP2A-Pep infusion into the VTA. Left: Representative images (upper row: low magnification images, scale bar 200 μm; lower row, high magnification images, scale bar 20 μm). Right: Quantification of fluorescence intensities (the data was normalized to the mean immunofluorescence value of control mice). The data represents the mean ± SD of 8–10 mice per condition. Statistical analysis: Two-way ANOVA with Tukey’s multiple comparison test (ns, *p* > 0.05). **(C)** GABA_B_ expression remained unaffected in the hippocampus of METH-addicted mice. Left: Representative images (upper row: low magnification images, scale bar 100 μm; lower row, high magnification images, scale bar 20 μm). Right: Quantification of fluorescence intensities (the data was normalized to the mean immunofluorescence value of control mice). The data represents the mean ± SD of 5–7 mice per condition. Statistical analysis: Two-way ANOVA with Tukey’s multiple comparison test (ns, *p* > 0.05). **(D)** GABA_B_ expression in the motor cortex remained unaffected in METH-addicted mice. Left: Representative images (upper row: low magnification images, scale bar 200 μm; lower row, high magnification images, scale bar 20 μm). Right: Quantification of fluorescence intensities (the data was normalized to the mean immunofluorescence of control mice). The data represents the mean ± SD of 7–8 mice per condition. Statistical analysis: Two-way ANOVA with Tukey’s multiple comparison test (ns, *p* > 0.05).

First, we analyzed whether GABA_*B*_ receptor expression in the VTA was reduced in METH-addicted mice. For this, we performed immunofluorescence staining for GABA_*B*_ receptors and the inactive control peptide (Ctrl-Pep) infused into the VTA of saline-injected control mice and METH-addicted mice. The area of the VTA containing neurons that displayed staining for the peptide was then analyzed for GABA_*B*_ receptor expression using antibodies directed against the GABA_*B*2_ subunit. As expected, we detected a considerable reduction of GABA_*B*_ receptor expression in the VTA in METH-addicted mice (Figure 2A). We then tested whether inhibiting the interaction of GABA_*B*_ receptors with PP2A would restore GABA_*B*_ receptor expression. Indeed, infusion of PP2A-Pep into the VTA of METH-addicted mice normalized GABA_*B*_ receptor expression as tested about 24 h after peptide application (Figure 2A). Infusion of PP2A-Pep into the VTA of non-addicted control mice had no effect on the expression of GABA_*B*_ receptors.

One of the main projection areas of the VTA involved in addictive phenotypes is the nucleus accumbens. Like the VTA, GABA_*B*_ receptor expression was significantly reduced in the nucleus accumbens but remained unaffected by infusion of PP2A-Pep into the VTA (Figure 2B). By contrast, GABA_*B*_ receptor expression in the hippocampus (Figure 2C) and motor cortex (Figure 2D) was not significantly affected in METH-addicted mice.

**FIGURE 3:**
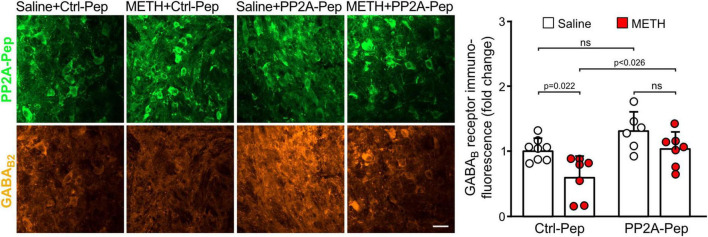
PP2A-Pep normalized GABA_B_ receptor expression in the somata of VTA neurons of METH-addicted mice. METH-addicted and control mice were infused into the VTA either with PP2A-Pep or the inactive Ctrl-Pep. About 24 h after peptide application, the mice were analyzed for GABA_B_ receptor expression in the soma of individual neurons that had taken up the peptide using GABA_B2_ antibodies. Left: Representative images displaying peptide uptake (green) and GABA_B_ receptor expression (orange) in VTA neurons (scale bar: 20 μm). Right: Quantification of fluorescence intensities (the data was normalized to the mean immunofluorescence value of Ctrl-Pep infused into the VTA of saline treated control mice). The data represents the mean ± SD of 15 neurons per mouse derived from 6–8 mice per condition. The symbols in the graph display the mean values of all neurons analyzed in a single mouse. Statistical analysis: Two-way ANOVA with Tukey’s multiple comparison test (ns, *p* > 0.05).

Next, we analyzed GABA_*B*_ receptor expression in the VTA on the cellular level. For this, we quantified immunofluorescence intensities for GABA_*B*2_ in the somata of peptide positive VTA neurons. As observed on the more global, VTA wide level, GABA_*B*_ receptor expression in the soma of individual VTA neurons was downregulated in METH-addicted mice (Figure 3). Infusion of PP2A-Pep into the VTA normalized GABA_*B*_ receptor expression in the somata of neurons in METH-addicted mice (Figure 3).

Taken together, these results indicate that GABA_*B*_ receptors were downregulated in the VTA and nucleus accumbens of METH-addicted mice. Infusion of PP2A-Pep into the VTA restored receptor expression to normal levels in the VTA but did not affect receptor expression in the nucleus accumbens.

### PP2A-Pep infusion into the VTA reduced METH-induced locomotor sensitization

**FIGURE 4:**
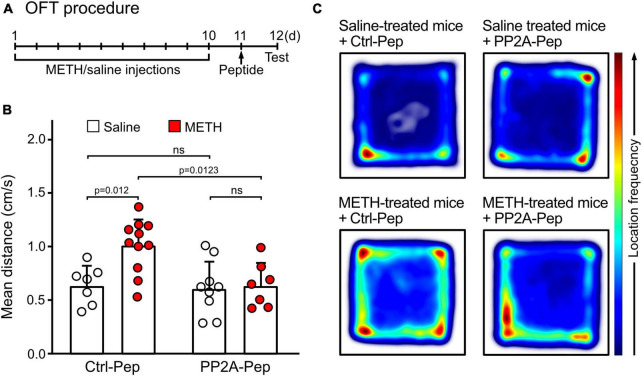
PP2A-Pep infusion into the VTA reduced METH-induced locomotor sensitization in the OFT. **(A)** Schematic outline of the OFT procedure. Mice received alternating METH and saline injections for 10 days to induce METH addiction. The control group received solely saline injections. One day after the last saline injection (day 11), the interfering peptide (PP2A-Pep) or the control peptide (Ctrl-Pep) was infused into the VTA via the implanted cannula. The next day, the mice were injected with a challenge dose of METH (1 mg/kg, i. p.) and immediately placed into the open-field test chamber to allow free exploration for 30 min. **(B)** METH-addicted mice exhibited increased locomotor activity, which was reduced to normal levels after PP2A-Pep injection into the VTA. Application of the control peptide (Ctrl-Pep) had no effect. The data represents the mean ± SD of 7–11 mice per condition. Statistical analysis: Two-way ANOVA with Tukey’s multiple comparison test (ns, *p* > 0.05). **(C)** Heat maps depicting the frequencies of locations visited by mice in the open field arena under the different experimental conditions (representative images).

Locomotor sensitization is a hallmark of psychostimulant-induced addiction (Tirelli et al., 2003). To test whether restoring GABA_*B*_ receptor expression in the VTA affects locomotor sensitization, mice were injected with METH and saline for ten days followed by infusion of PP2A-Pep or Ctrl-Pep into the VTA. Locomotor sensitization was then tested the next day in the open field arena (Figure 4A). METH-addicted mice showed increased locomotor activity, which was reduced to levels of non-addicted, saline injected, control mice that were infused with the non-active control peptide (Ctrl-Pep, Figures 4B, C). Infusion of PP2A-Pep into the VTA of non-addicted control mice did not affect locomotor activity (Figures 4B, C). Thus, PP2A-Pep effectively inhibited increased locomotor in METH-addicted mice.

### PP2A-Pep infusion into the VTA of METH-addicted mice reduced drug seeking behavior

**FIGURE 5:**
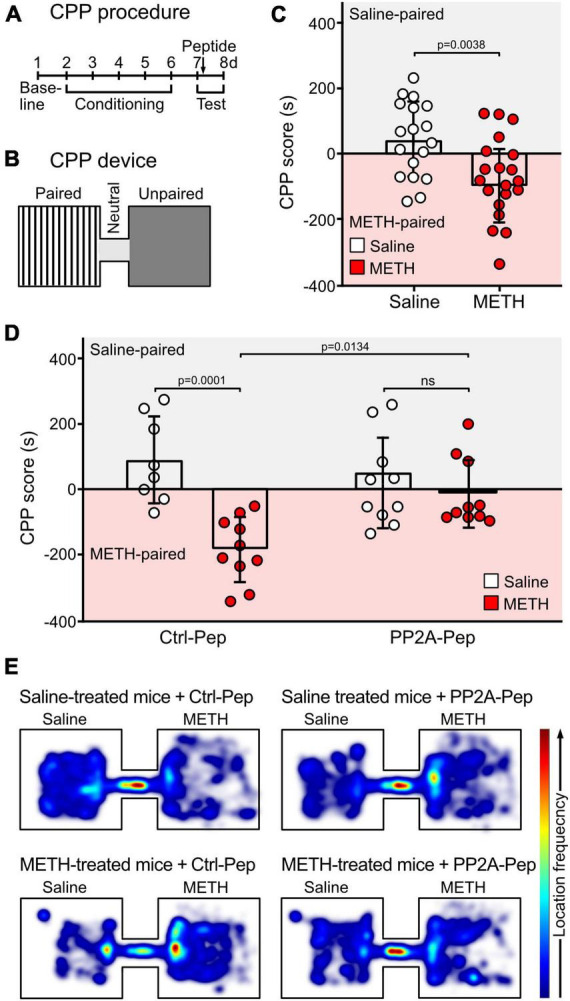
Injection of PP2A-Pep into the VTA of METH-addicted mice reduced drug seeking behavior as tested by CPP. **(A,B)** Schematic outline of the CPP procedure **(A)** and the CCP device **(B)**. CPP was conducted in a device consisting of two visually distinct plexiglass compartments connected through a short gray tunnel. The CPP procedure included three phases: habituation and baseline preference (day 1), conditioning phase (days 2–6) and preference tests (days 7 and day 8). During the conditioning phase (day 2–6), mice received METH injections (2 mg/kg, i. p.) in the initially non-preferred compartment and access was restricted to this compartment. After 6 h, mice were injected with saline in their initially preferred compartment. The control group received the same procedure, but mice were injected with two saline doses. On the test day (day 7, 24 h after the last conditioning trial), the preference test was performed. One hour after the test, mice were microinfused either with PP2A-Pep or Ctrl-Pep via the implanted cannula and tested a second time the next day (day 8). **(C)** METH-conditioned mice showed increased preference for the METH-paired chamber on the first test day. The data represents the mean ± SD of 18–20 mice per condition. Statistical analysis: Two-tailed unpaired *t*-test. **(D)** Infusion of PP2A-Pep into the VTA after the first test diminished the preference for the METH-paired chamber in METH-addicted mice on the second test day. Application of the control peptide (Ctrl-Pep) had no effect. The data represents the mean ± SD of 8–10 mice per condition. Statistical analysis: Two-way ANOVA with Tukey’s multiple comparison test (ns, *p* > 0.05). **(E)** Heat maps depicting the frequencies of locations visited in the CPP chambers by mice under the different experimental conditions (representative images).

The effect of PP2A-Pep on drug seeking behavior of METH-addicted mice was evaluated in the CPP test (Bardo and Bevins, 2000; McKendrick and Graziane, 2020). For the CPP test, we used a biased design in which the preference of the mice for one of the two compartments was determined before starting the conditioning phase (a schematic outline of the device and the experimental procedure is shown in Figures 5A, B). During the conditioning phase, the METH group received METH injections in the initially non-preferred compartment, followed by saline injections 6 h later in the opposite compartment. Mice in the saline control group received injections in the preferred compartment, followed by a second saline injection 6 h later in the opposite compartment. One day after the conditioning phase (day 7), mice were subjected to the CPP test and 1 h thereafter the mice were microinfused either with PP2A-Pep or Ctrl-Pep via the implanted cannula and returned to the home cage. A second preference test was performed the next day. As expected, METH-conditioned mice spent more time in the METH-paired compartment, indicating drug seeking behavior (Figure 5C). Infusion of PP2A-Pep into the VTA of METH-conditioned mice reduced the drug seeking behavior as the mice explored both compartments almost equally. Infusion of the inactive control peptide into the VTA of MET-conditioned mice was without effect as was infusion of PP2A-Pep into the VTA of saline control mice. These results indicate that a single administration of PP2A-Pep into the VTA of METH-addicted mice diminishes drug seeking behavior.

## Discussion

This study indicates that repeated METH treatment in mice downregulates GABA_B_ receptor expression in the VTA. Infusion of an interfering peptide that inhibits the interaction of GABA_B_ receptors with PP2A into the VTA of METH-treated mice restored the expression of GABA_B_ receptors. This diminished METH-induced locomotor sensitization and drug seeking behavior.

Excessive neuronal excitation triggers the downregulation of GABA_B_ receptors by increasing their lysosomal degradation on the expense of recycling the receptors back to the plasma membrane (Guetg et al., 2010; Maier et al., 2010; Terunuma et al., 2010). Two key steps are involved in this pathway: CaMKIIβ-mediated phosphorylation of the GABA_B1_ subunit at Ser867 (Guetg et al., 2010; Zemoura et al., 2019; Balakrishnan et al., 2023) and PP2A-dependent dephosphorylation of the GABA_B2_ subunit at Ser783 (Terunuma et al., 2010; Hleihil et al., 2022). Elevated intracellular Ca^2+^ levels due to enhanced neuronal activity activates CaMKIIβ to induce phosphorylation of Ser867 in GABA_B1_ (Maier et al., 2010). Recent evidence suggests that CaMKIIβ not directly phosphorylate GABA_B1_ but activates ERK1/2 which in turn phosphorylate Ser867 and the adjacent Thr872 (Bhat et al., 2023). Phosphorylation of GABA_B1_ is required to enable K63-linked ubiquitination of the receptor by the E3-ubiquitin ligase MIB-2, which serves as a signal for their lysosomal degradation (Zemoura et al., 2019). In addition to CaMKIIβ, activation of PP2A under these conditions dephosphorylates Ser783 in GABA_B2_. This inhibits fast recycling of the receptors and directs them into the degradation pathway controlled by CaMKIIβ (Terunuma et al., 2010; Hleihil et al., 2022).

In the present study we found that in most neurons of the VTA analyzed, GABA_B_ receptors were downregulated after repetitive METH treatment in mice. Likewise, we found a similar reduction of GABA_B_ receptors in the nucleus accumbent, a main projection area of the VTA neurons associated with drug addiction. This finding is well supported by a recent study indicating reduced GABA_B_ receptor expression in the nucleus accumbens of cocaine-addicted rats involving CaMKII dependent phosphorylation of the receptors (Lu et al., 2023). By contrast, GABA_B_ receptor expression in the hippocampus and motor cortex remained unaffected, reassuring that the changes in immunostaining we detected were not caused by factors unrelated to the treatment.

In all our previous studies, a loss of GABA_B_ receptor immunofluorescence staining always correlated with a similar reduction of receptor protein signals in western blots and with diminished GABA_B_ receptor mediated inhibition (see e.g., Zemoura et al., 2019; Hleihil et al., 2021, 2022; Bhat et al., 2022; Balakrishnan et al., 2023). Therefore, it is reasonable to assume that the reduction in GABA_B_ receptor immunofluorescence staining observed in this study translates into diminished GABA_B_ receptor mediated inhibition. The METH-induced downregulation of GABA_B_ receptors we observed in VTA neurons is well supported by diminished GABA_B_ receptor-mediated inhibition reported in the VTA (Arora et al., 2011; Padgett et al., 2012; Sharpe et al., 2015; Munoz et al., 2016). So far, two mechanisms have been reported to cause the reduction of GABA_B_ receptor-mediated inhibition in the VTA. First, in GABAergic neurons, the receptors were downregulated from the cell surface involving dephosphorylation of GABA_B2_ Ser-783 after a single injection of METH (Padgett et al., 2012). Second, in dopaminergic neurons, the postsynaptic GABA_B_ receptor effectors, the GIRK channels, were downregulated instead of the receptors (Arora et al., 2011; Munoz et al., 2016). However, our data suggests a more widespread downregulation of GABA_B_ receptors in VTA neurons than just their selective reduction in GABAergic neurons since only about 35% of neurons in the VTA are GABAergic (∼60% are dopaminergic and ∼5% glutamatergic) (Nair-Roberts et al., 2008; Ma et al., 2023). Further investigations are required to address the proportion of GABA_B_ receptor downregulated in the different neuronal populations of the VTA. In addition, it would be interesting to analyze in detail whether solely cell surface GABA_B_ receptors are reduced as previously reported after a single METH injection (Padgett et al., 2012) or more globally as we observed after repeated injections. However, infusion of PP2A-Pep into the VTA restored GABA_B_ receptor expression in VTA neurons, supporting the view that the observed downregulation of GABA_B_ receptors in METH-addicted mice is mediated by the PP2A/CaMKII pathway described above. The restoration of GABA_B_ receptors in the VTA also reduced addictive behaviors in METH-addicted mice, suggesting that a globally restored inhibitory control by GABA_B_ receptors in the VTA diminishes enhanced dopaminergic activity in the nucleus accumbens. Interestingly, restored GABA_B_ receptor expression in the VTA did not normalize reduced GABA_B_ receptor levels in the nucleus accumbens during the time frame of our analysis. Cocaine treatment most likely downregulates GABA_B_ receptor expression in the nucleus accumbens by the same mechanism as METH treatment in the VTA (Lu et al., 2023). This suggests that simply reducing dopaminergic activity to the nucleus accumbens is not sufficient to normalize the aberrant trafficking of the receptors. It would be interesting to analyze the effect of PP2A-Pep on GABA_B_ receptor expression in the nucleus accumbens and on addictive phenotypes of METH addicted mice.

Repeated psychostimulant administration in rodents leads to locomotor sensitization, a phenotype that is triggered by enhanced dopamine release within the VTA and nucleus accumbens. Re-exposure to even a small quantity of the psychostimulants in the addicted subject, induces a strong activity in the VTA (Tirelli et al., 2003; Steketee and Kalivas, 2011). Accordingly, we observed a significant increase in locomotor activity upon injection of a challenge dose of the drug in METH-addicted mice. Interestingly, a single injection of PP2A-Pep into the VTA was sufficient to reverse the enhanced locomotor activity, most likely due to increased tonic inhibition mediated by the restored GABA_B_ receptor expression in the VTA. This finding is in line with previous studies showing that the GABA_B_ receptor agonist baclofen inhibits locomotor sensitization (Bartoletti et al., 2004; Leite-Morris et al., 2004; Cedillo and Miranda, 2013). Furthermore, selective deletion of GABA_B_ receptors from VTA dopaminergic neurons enhanced the locomotor sensitization to psychostimulants (Edwards et al., 2017), illustrating the importance of GABA_B_ receptor mediated inhibition for controlling locomotor sensitization.

It is believed that drug addiction is a form of Pavlovian conditioning, establishing long-term memories to stabilize drug associated behaviors. In the CPP experiment, associations are formed between a rewarding stimulus (e.g., METH) and a contextual environment (Tzschentke, 2007; McKendrick and Graziane, 2020). Re-exposure to drug-associated cues triggers memory recall, making these memories labile and sensitive to manipulations (Tronson and Taylor, 2007; Bellfy and Kwapis, 2020). In our CPP test, we aimed to interfere with METH-associated memories causing drug seeking by disturbing memory reconsolidation. This was done by infusing PP2A-Pep into the VTA 1 h after re-exposing the mice to the CPP test and repeating the CPP test after 24 h. This schedule was chosen since it is relevant in view of the development of a potential therapeutic application. Restoration of GABA_B_ receptor expression in the VTA by PP2A-Pep after memory retrieval inhibited drug seeking behavior in the re-test session the next day. This finding suggests that METH associated memory reconsolidation has been disturbed by enhancing the expression of GABA_B_ receptors in the VTA.

However, our CPP protocol cannot rule out the possibility that the interfering peptide might additionally facilitate memory extinction, a process which is tightly associated with psychostimulant withdrawal (Myers and Carlezon, 2010). It is believed that memory extinction is an active learning process, where a newly learned memory is competing with the already consolidated one to weaken it. Memory consolidation and extinction have overlapping cellular and molecular mechanisms (Myers and Carlezon, 2010; Liu et al., 2019), making it difficult in our experimental set-up to discriminate among these two pathways. However, in view of the close timing of PP2A application 1 h after memory retrieval we favor memory reconsolidation to be affected by PP2A-Pep in the first place.

We expect that PP2A-Pep is associated with negligible adverse side effects because it selectively inhibits the interaction of GABA_B_ receptors with PP2A and does not appreciably affect GABA_B_ receptor expression in healthy control mice. Therefore, side effects observed after global activation the receptors with the agonist baclofen such as sedation, dizziness, fatigue, insomnia, headache, paresthesia, tinnitus, and restlessness (Rolland et al., 2018) are unlikely to occur. In addition, because the peptide sequence of PP2A-Pep derived from the C-terminal domain of the GABA_B2_ subunit is unique to GABA_B_ receptors, we expect no off-target effects involving other receptor systems. In principle, binding of PP2A-Pep to PP2A could downregulate the expression levels of PP2A, which would affect a variety of signaling pathways involving protein dephosphorylation by PP2A. However, we previously showed that PP2A-Pep did not affect expression of PP2A (Hleihil et al., 2022).

In conclusion, restoring GABA_B_ receptor expression in VTA neurons of METH-addicted mice by inhibiting PP2A-mediated downregulation of the receptors inhibited locomotor sensitization and drug seeking behavior. This finding indicates that interfering with disease-relevant protein-protein interactions might be a promising alternative approach toward the development of a selective and efficient therapeutic intervention.

## Data availability statement

The datasets analyzed for this study have been deposited at ZENODO.org and are publicly available (doi: 10.5281/zenodo.10554456). Due to their large size, raw images and behavioral recordings can be made available upon reasonable request to the corresponding author.

## Ethics statement

The animal study was approved by the Veterinary office of the Canton of Zurich, Waltersbachstrasse 5, CH-8006 Zurich (license ZH164/2020). The study was conducted in accordance with the local legislation and institutional requirements.

## Author contributions

MH: Conceptualization, Data curation, Formal Analysis, Writing – original draft, Writing – review & editing, Investigation, Methodology. DB: Conceptualization, Data curation, Formal Analysis, Writing – original draft, Writing – review & editing, Funding acquisition, Project administration, Resources, Supervision.
